# A Deep Learning-Based Unbalanced Force Identification of the Hypergravity Centrifuge

**DOI:** 10.3390/s23083797

**Published:** 2023-04-07

**Authors:** Kuigeng Lin, Yuke Li, Yunhao Wu, Haoran Fu, Jianqun Jiang, Yunmin Chen

**Affiliations:** 1 Department of Civil Engineering, Zhejiang University, 866 Yuhangtang Road, Hangzhou 310058, China; 2 NetEase Yidun AI Lab, Hangzhou 310051, China

**Keywords:** hypergravity centrifuge, unbalanced force identification, deep learning

## Abstract

Accurate and quantitative identification of unbalanced force during operation is of utmost importance to reduce the impact of unbalanced force on a hypergravity centrifuge, guarantee the safe operation of a unit, and improve the accuracy of a hypergravity model test. Therefore, this paper proposes a deep learning-based unbalanced force identification model, then establishes a feature fusion framework incorporating the Residual Network (ResNet) with meaningful handcrafted features in this model, followed by loss function optimization for the imbalanced dataset. Finally, after an artificially added, unbalanced mass was used to build a shaft oscillation dataset based on the ZJU-400 hypergravity centrifuge, we used this dataset to train the unbalanced force identification model. The analysis showed that the proposed identification model performed considerably better than other benchmark models based on accuracy and stability, reducing the mean absolute error (MAE) by 15% to 51% and the root mean square error (RMSE) by 22% to 55% in the test dataset. Simultaneously, the proposed method showed high accuracy and strong stability in continuous identification during the speed-up process, surpassing the current traditional method by 75% in the MAE and by 85% in the median error, which provided guidance for counterweight and guaranteed the unit’s stability.

## 1. Introduction

Hypergravity centrifugal test techniques have widely been used in slope and high dam engineering, geotechnical seismic engineering, deep sea engineering, deep underground engineering, geological process research, advanced material manufacture, and other research fields. However, with the increasing capacity and speed of the hypergravity centrifuge, the unbalanced force also increases rapidly. The unbalanced force is the cyclic disturbance caused by the mass-radius product difference at the two ends of the rotational arm. The unbalanced force is attributed to counterweight error, inherent processing error, inhomogeneous material, or onboard test models’ deformation and movement. The unbalanced force is the primary problem that affects centrifuges’ service life and causes safety accidents. It is also an essential factor that causes centrifuge vibrations to affect test accuracy. Therefore, identifying the unbalanced force in hypergravity centrifuges under different operating conditions is critical.

For unbalanced force in rigid rotor systems, such as the hypergravity centrifuge, the commonly used identification and diagnosis methods can be divided into four major categories: the influence coefficient method, model-based unbalance identification method, parameter identification-based unbalance identification method, and the unbalance fault diagnosis method based on intelligent algorithms. Particularly, while the influence coefficient method needs to start and stop the rotor system several times using trial weights, it utilizes sensors to collect the unbalanced response of the rotor system. In this method, the balance correction mass required for the next operation process is notably adjusted according to the influence coefficient of the unbalanced mass on the response until the vibration value at the balancing speed meets the requirements. The dynamic balance method applicable to multiple balancing surfaces and multiple rotating speeds was first developed by Goodman et al. [1] through introducing a least-squares solution of the balance equation to the initial prototype of the influence coefficient theory. Based on Goodman, many studies have expanded and validated the use of the influence coefficient method. For example, Kang Y et al. [2] analyzed and verified that the efficiency and accuracy of dynamic balance could be improved by optimizing the position of the vibration sensor and balancing surface. Lei W et al. [3] decomposed the axial trajectory into forward and backward precession by arranging two mutually perpendicular displacement transducers and replacing the conventional unbalanced response with the amplitude and phase of the forward precession, improving the accuracy of the influence coefficient method.

The model-based unbalance identification method combines the measured unbalanced response of the rotor system with a rotor dynamic model to identify the rotor system’s unbalance. With the help of the rotor dynamic model, the rotor system’s unbalanced magnitude, phase, position, and other parameters can be obtained. Edwards S et al. [4] measured the response of a rotor system and then established the transfer matrix for the magnitude and phase of the unbalance, thereby developing a model-based unbalance identification approach. Since their method could not detect the eccentric and torsional vibrations of different structural forms simultaneously, modal analysis techniques assisted by the finite element method (FEM) or Fourier transform method have been applied to improve accuracy. In another study, Richard Markert et al. [5] used a time-domain least-squares method to compare the measured equivalent loads with those calculated from rotor dynamics and investigated the effect of factors, such as measurement noise, measurement location, and the number of modes on the identification results. Then, Sudhakar et al. [6] evaluated the residuals between the measured rotor’s response and the FEM simulated response, optimizing the model-based unbalance identification method.

Contrastively, the parameter identification-based unbalance identification method considers the calculation of unbalanced force from unbalanced responses as an inverse problem. Pennacchi et al. [7,8] significantly developed a model-based unbalance identification by considering it a parameter estimation problem. Then, they compared and analyzed the effects of various estimation methods, parameters, and other factors on the identification accuracy and robustness. Unfortunately, the model-based unbalance identification method is ill-posed when the response measurement location is less than the unbalance fault location. Hence, Chatzisavvas et al. [9] proposed an imbalance identification method combined with regularization techniques to achieve a more accurate identification without priori information.

The unbalance fault diagnosis method based on intelligent algorithms regards unbalance as a typical fault. Accordingly, this approach extracts fault characteristics from response data and uses intelligent algorithms for diagnosis and identification based on fault mechanism analysis. For example, Hübner et al. [10] proposed a support vector machine (SVM)-based unbalance fault diagnosis method using current and voltage data from a condition monitoring system in a wind turbine to achieve a classification diagnosis of an unbalanced mass. You L et al. [11] proposed a rotor unbalance fault classification method using an improved shuffled frog leaping algorithm to optimize the SVM parameters. Tong R et al. [12] validated the effectiveness and superiority of the adaptive weighted kernel extreme learning machine algorithm in the blade icing detection of wind turbines through three benchmark cases. Chen J et al. [13] developed a long short-term memory neural network (LSTM) model to extract fault signal features. Similarly, many studies have also emerged using Markov Transition Field (MTF) [14] and Gramian Angular Field (GAF) [15] for converting one-dimensional time-domain data into two-dimensional images to exploit widely developed image recognition techniques. In these studies, various intelligent algorithms have been used in fault diagnosis, including machine learning methods: SVM [16], Extreme Tree Regression (ETR) [17], and eXtreme Gradient Boosting algorithm (XGBoost) [18]; and deep learning models: Convolution Neural Network (CNN) [19], Deep Belief Networks (DBN) [20], GoogleNet [21], MobileNet [22], Deep Residual Networks (ResNet) [23], and Squeeze-and-Excitation Networks (SEnet) [24].

Among the above methods, the influence coefficient method has been widely used in field dynamic balance because it is easy to use without the deep knowledge requirements of rotor structure and dynamic characteristics. However, hypergravity test models, such as the geotechnical model, fail after one centrifugal process, making a trial weight process inapplicable. Conversely, although the model-based and parameter identification-based unbalance identification methods have the advantage of identifying the unbalance without trial weight processes, it is impossible to establish accurate simulation models due to the complex structure of hypergravity centrifuges and the unknown bearing parameters. Therefore, the model-based and parameter identification-based unbalance identification method cannot be applied directly to the unbalanced force identification of hypergravity centrifuges. Furthermore, while deep learning-based fault diagnosis methods can reduce the impact of handcrafted features obtained from feature extraction methods based on a priori knowledge, the current unbalance fault diagnosis can only qualitatively determine the unbalanced state, making it impossible to meet the quantitative identification demand of unbalanced force in hypergravity centrifuges. To this end, a method to quantitatively identify the balance state of a hypergravity centrifuge without trial weight processes should be established.

Based on the above facts, this study aims at an unbalanced force identification method dependent on deep learning for hypergravity centrifuges. We first introduce the proposed deep learning-based unbalanced force identification approach and related methodologies, including the GAF method, ResNet algorithm, and Tweedie loss for optimizing the identification performance in Section 2. Then, an unbalanced response dataset is obtained by testing in the ZJU-400 hypergravity centrifuge. The description of the test and data are detailed in Section 3. The experimental setup and evaluation methods of this paper are detailed in Section 4. After the model is constructed and optimized based on the given training data, we compare the proposed approach with several widely used benchmark approaches to analyze the performance of unbalanced force identification. A demonstration of an entire speed-up process of ZJU-400 validates the proposed method and suggests potential applications in unbalanced force identification. The comparative discussion and performance evaluation are introduced in Section 5. Finally, the conclusion is presented in Section 6.

## 2. Deep Learning-Based Identification Methodology

### 2.1. GAF: Encoding Time Series as Images

A method for converting one-dimensional time series into two-dimensional images is often employed to use the widely developed deep learning method in image recognition. Accordingly, the GAF [25] method first aligns the time-domain data to the same length, rescales the values to [−1, 1], and then recodes the time-domain data using polar coordinates. The encoded time series can thus be converted into two-dimensional images using Gram Angular Sum Field (GASF) and Gram Angular Difference Field (GADF). Furthermore, GAF mainly has the advantage of temporal correlation, with the image pixel matrix containing information about the values and angles of the original series in the main diagonal. Consequently, the time series can be reconstructed based on the high-level features learned by the deep neural network.

### 2.2. ResNet: Convolutional Neural Network (CNN) Based on Residual Learning

ResNet, which solves the gradient disappearance/gradient explosion problem caused by increasing the number of deep network layers, is developed based on residual learning and CNN. It is a deep neural network comprising multiple residual building blocks (RBB) stacked on each other. By adding shortcut connections across the convolution layer, RBB allows the network to span some intermediate layers, directly passing information to deeper layers. Such shortcut connections help to optimize the trainable parameters in error backpropagation without adding additional parameters and computational complexity, avoiding the problem of gradient disappearance or gradient explosion and helping to construct network structures with more layers. Notably, ResNet at different network depths can be obtained by stacking different amounts of RBB. Thus, depending on the number of RBB, examples of ResNet structures commonly used at different depths are ResNet-18, ResNet-34, ResNet-50, and ResNet-101.

### 2.3. Tweedie Loss: The Optimized Loss for an Imbalanced Dataset

For regression problems, the mean absolute error (MAE) or mean squared error (MSE) is often used as a model’s loss or evaluation function. The potential hypothesis for selecting these two errors as loss functions is that the probability density function of the sample label corresponds to the normal distribution. However, in some usage scenarios, such as in actuarial science, widely available semi-continuous data consists mainly of zero and positive continuous data. This type of distribution is called the Poisson–Gamma composite distribution or Tweedie composite Poisson distribution.

If a random variable Y obeys the Tweedie composite Poisson distribution, it can be expressed as a random expression as follows:

(1) $$Y=\sum_{i=1}^{P}X_{i},$$

where P is a random variable conforming to the Poisson distribution with a mean parameter λ, and the variable’s value is denoted as $X_{i}$. For a given P, $X_{i}(1\leq i\leq P)$ should be independent, have identical distributions, and obey a Gamma distribution with mean αγ and variance $\alpha \gamma^{2}$.

Since the Tweedie composite Poisson distribution is a particular exponential dispersion distribution [26], the probability density function of its exponential dispersion distribution can be expressed as

(2) $$p(y;\theta ,\phi )=a(y,\phi )\exp\left\{\frac{y\theta -\kappa (\theta )}{\phi }\right\},$$

where a(⋅) and κ(⋅) are known functions, θ is a parameter defined on ℝ, ϕ is the dispersion parameter, and ϕ>0.

According to the properties of the exponential dispersion distribution, the following expressions also hold:

(3) $$E(Y)=\mu =\kappa^{\prime }(\theta ),$$

(4) $$\mathrm{Var}(Y)=\phi \kappa^{\prime \prime }(\theta ),$$

(5) $$\mathrm{Var}(Y)=\phi \mu^{\rho },$$

where $\kappa^{\prime }(\theta )$ and $\kappa^{\prime \prime }(\theta )$ are the first and second derivatives of θ, respectively.

Consequently, the value of the power parameter ρ determines the distribution of the random variable Y, obeying the Tweedie composite Poisson distribution when 1<ρ<2, the Poisson distribution when ρ=1, and the Gamma distribution when ρ=2. In such cases, the probability density function of the random variable Y can be expressed as

(6) $$f_{Y}(y\left|\theta ,\phi \right.)=a(y,\phi )\exp\left\{\frac{1}{\phi }\left(y\frac{\mu^{1-\rho }}{1-\rho }-\frac{\mu^{2-\rho }}{2-\rho }\right)\right\},$$

the log-likelihood function of the Tweedie composite Poisson distribution can be expressed as

(7) $$\log f_{Y}(y\left|\theta ,\phi \right.)=\frac{1}{\phi }\left(y\frac{\mu^{1-\rho }}{1-\rho }-\frac{\mu^{2-\rho }}{2-\rho }\right)+\log a(y,\phi ),$$

and the log-likelihood function for each sample of the dataset can be expressed as

(8) $$L(\theta )=\sum_{i=1}^{N}\log f_{Y}(y\left|\theta ,\phi \right.)\;\propto \sum_{i=1}^{N}\left(y_{i}\frac{\mu_{i}^{1-\rho }}{1-\rho }-\frac{\mu_{i}^{2-\rho }}{2-\rho }\right).$$

Accordingly, a training objective then maximizes the likelihood function, i.e., minimizes Tweedie loss:

(9) $$\mathrm{Tweedie}\;\mathrm{loss}=\frac{1}{P}\sum_{i=1}^{N}-\left(y_{i}\frac{\mu_{i}^{1-\rho }}{1-\rho }-\frac{\mu_{i}^{2-\rho }}{2-\rho }\right),$$

where $y_{i}(1\leq i\leq P)$ is the actual value and $\mu_{i}(1\leq i\leq P)$ is the regression value.

**Figure 1:**
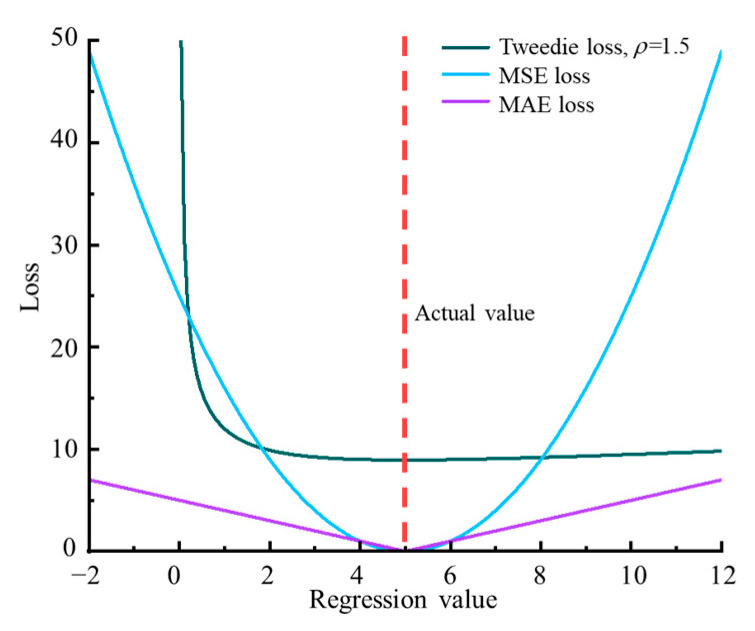
Comparison of Tweedie loss with MAE and MSE losses.

Figure 1 compares the Tweedie loss (ρ=1.5) with MAE and MSE losses for different regression values with an actual value of 5. Since MSE loss was sensitive to the outliers that deviated from the actual value, the loss gradient varied with error. Conversely, since MAE loss was more robust to outliers, the loss gradient was fixed at ±1. Since the Tweedie loss is nonzero and correlates with the actual value when the regression value is consistent with the actual value, it can only be used for regression problems where the actual values exceed 0. Additionally, the Tweedie loss would have a more severe penalty for regression values smaller than actual values, which is close to scenarios, such as insurance prediction.

### 2.4. Deep Learning-Based Unbalanced Force Identification Model

**Figure 2:**
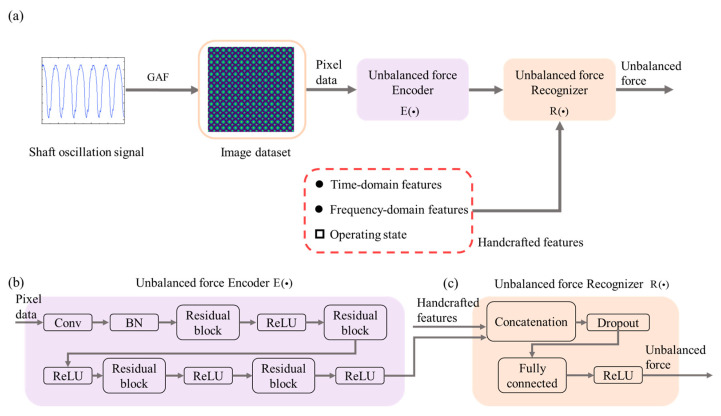
The architecture of the unbalanced force identification model. (**a**) A neural network based on the feature fusion framework. (**b**) The detailed architecture of the encoder. (**c**) The detailed architecture of the recognizer.

Finally, we propose a feature fusion framework to combine the features learned by the deep structures with some meaningful handcrafted features to improve the performance of unbalanced force identification and fully use all available information. Figure 2 shows the unbalanced force identification model for the hypergravity centrifuge proposed in this paper. First, the signal segment was transformed into two-dimensional images by GAF, after which we fed the image into an encoder built using a ResNet network for feature learning. Next, we extracted signal and state features based on a priori experience as handcrafted features, and then we concatenated the handcrafted features and the features learned through ResNet to form a complete feature set to be input into the recognizer composed of fully connected layers to identify the unbalanced forces.

Unbalanced force identification in hypergravity centrifuges is a typical regression problem. Moreover, similar dataset imbalance problems in the training dataset are encountered daily. Therefore, this paper investigated the recognition effect of using Tweedie loss on an imbalanced dataset. We used the Tweedie loss function to calculate and back-propagate the training data loss, including the actual and recognized values, to generate error gradients for each layer in the encoder/recognizer. The adaptive learning rate optimization approach is adopted for each parameter in the model [27]. Considering the overfitting problem in deep learning models, appropriate regularization techniques should be adopted to make the model performance of the training and test datasets close to each other. Hence, this paper used the dropout technique [28] to solve this problem. First, dropout layers were added to the recognizer, after which the outputs of part of the hidden layers were randomly blocked according to the set probability. As a result, these neurons did not affect the forward propagation during the model’s training. During testing, however, the dropout layer was kept off, involving all hidden layers in the computation.

## 3. Data Description

### 3.1. Case Study: The ZJU-400 Hypergravity Centrifuge

The unbalanced response of the hypergravity centrifuge was obtained by testing via a ZJU-400 hypergravity centrifuge. The ZJU-400 hypergravity centrifuge is designed for a maximum operating capacity of 400 *g*·t (product of centrifugal acceleration and mass of load), a maximum centrifugal acceleration of 150 *g*, and a maximum effective rotational radius of 4.5 m. Notably, the centrifugal acceleration (in units of gravitational acceleration *g*) at the bottom of the basket is a critical parameter in designing a hypergravity test model that can indicate the rotating speed status of the unit, e.g., the maximum centrifugal acceleration 150 *g* corresponds to a rotating speed of 174 rpm for ZJU-400. Accordingly, the parameter ψ is called centrifugal acceleration for short in the following sections.

We provided unbalanced forces by artificially adding mass at the bottom of one side of the hanging basket when the centrifuge was in balance. Afterward, we simulated the operating load by placing weight blocks on both sides of the hanging basket. The unbalanced force in each experimental condition was determined by the amount of artificially added mass and the centrifugal acceleration via the expression below:

(10) F=mψ,

where m is the artificially added mass, ψ is the centrifugal acceleration at the bottom of the hanging basket of the hypergravity centrifuge (expressed as $\psi =4\pi^{2}f^{2}r$), f is the rotating frequency, and r is the nominal rotational radius. In the design and utilization of hypergravity centrifuges, force is expressed by the unit of ton-force (tf), defined as the weight of one ton due to standard gravity.

**Figure 3:**
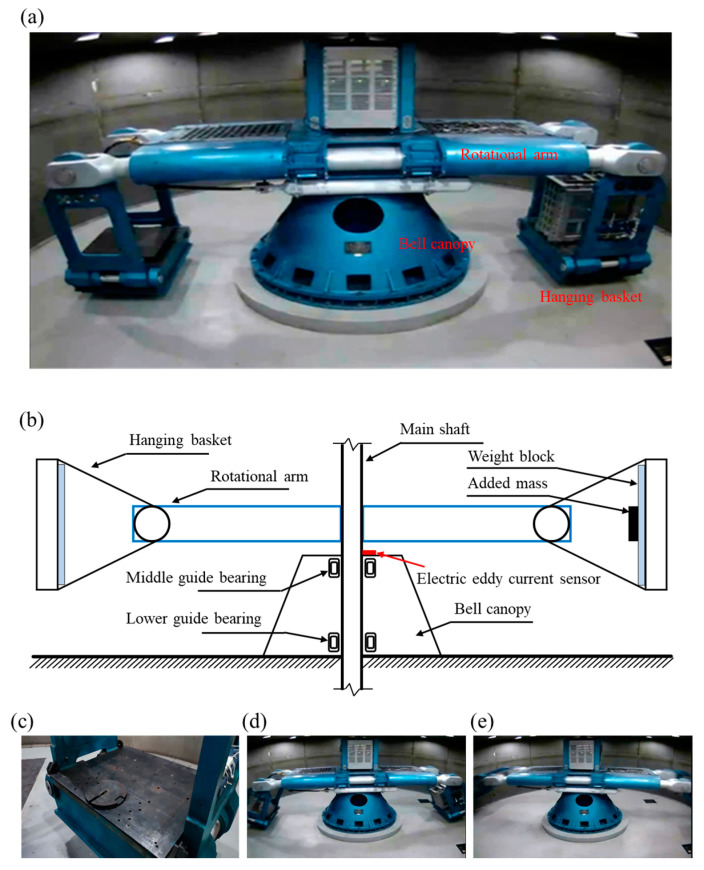
The ZJU-400 hypergravity centrifuge’s structure. (**a**) The main structure. (**b**) Schematic showing the rotor system and sensor arrangement position. (**c**) Artificially added mass before operation. (**d**) Operating condition at low centrifugal acceleration. (**e**) Operating condition at high centrifugal acceleration.

Several experimental groups were designed according to the centrifugal acceleration range, the ZJU-400 hypergravity centrifuge load range, and the maximum tolerated unbalance force. Multiple centrifugal acceleration test groups were set between 0 and a maximum centrifugal acceleration of 150 *g*. The artificially added unbalanced masses were 20 kg, 40 kg, 60 kg, 100 kg, and 200 kg. The operating load was set to no load (0 t) and large load (2 t). Table 1 and Table 2 show the experimental groups and the unbalanced force included in the training and test datasets. Figure 3a shows the structure of the ZJU-400 hypergravity centrifuge. In the experiment, after installation of the eddy current sensor above the bell canopy (Figure 3b), we placed the artificially added mass at the bottom of hanging basket (Figure 3c), and then gradually increased the rotating speed of the hypergravity centrifuge (Figure 3d,e). After the design acceleration was reached, the radial oscillation signal of the shaft was collected through the eddy current sensor. Each group of experimental data was tested for 4 min at a sampling frequency of 1000 Hz.

**Figure 4:**
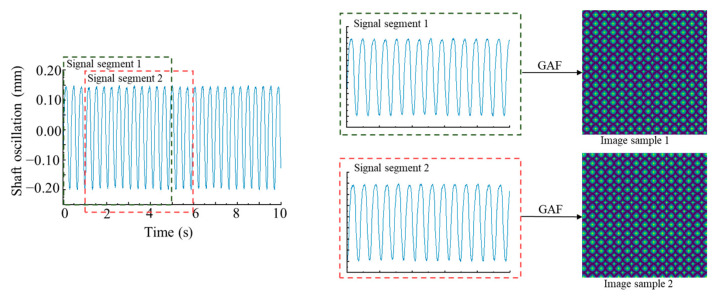
An example of data segmentation.

In the study of this paper, 200 signal samples were first obtained from the shaft oscillation signals under each independent test group to avoid randomness, specificity, and unsteadiness signal influences on the identification results. Next, the data were divided into the training dataset (about 65%) and the test dataset (about 35%) according to the application requirements and the actual data distribution. Each signal sample was a measured shaft oscillation signal segment with a sampling duration of 5 s that can be obtained in a partially overlapping form through a sliding window. Since the actual signal was concentrated in the low-frequency region without any high-frequency signal, the original signal was resampled at 100 Hz to save computational effort. Subsequently, we generated images using GADF [25] for the resampled data at a resolution of 500 × 500. Figure 4 shows an example of sample segmentation. Finally, the training dataset was expanded by adding Gaussian white noise to the original time-domain signal to enhance the noise resistance of the model. Three sets of white noise were added, and the signal-to-noise ratios of the signals are 25 dB, 30 dB, and 35 dB, respectively.

**Figure 5:**
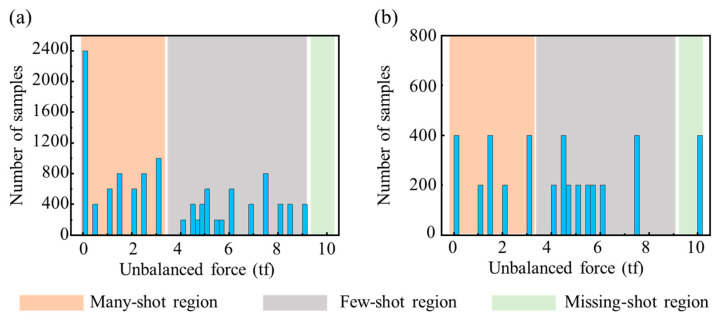
The distribution of the unbalanced force labels. (**a**) Training dataset. (**b**) Test dataset.

Figure 5 shows the distribution of the unbalanced force labels for the training and test datasets. The distribution shows an approximate long-tailed distribution with numerous small, unbalanced force labels and a few sizable, unbalanced force labels, indicating that the dataset was imbalanced. This dataset imbalance can seriously affect the performance of deep learning-based models. Therefore, labels were divided into three regions according to the number of samples [29]: the Many-shot region for those below 4 tf, the Few-shot region for those with 4–9 tf, and the Missing-shot region for those above 9 tf. Accordingly, while evaluating the identification errors, the label distribution in the test dataset was set up to approximate a uniform distribution and avoid the particularity of the test dataset due to the label distribution problem.

### 3.2. Handcrafted Features and Correlation Analyses

In the fault diagnosis of a rotor system based on vibration signals, extracting the vibration signal’s time- and frequency-domain features is often necessary. While commonly used time-domain features include root mean square value (RMS), peak value, kurtosis, standard deviation (STD), etc., typical frequency-domain features include spectral magnitude, frequency band energy, center frequency, etc. Notably, when a rotor is subjected to unbalanced forces, its vibration response is mainly characterized by forced vibrations dominated by rotating frequency, causing the waveform of the shaft oscillation signal to be close to a sinusoidal signal in the time domain (Figure 4). The rotating, double, and triple frequencies can then be observed in the frequency spectrum. Subsequently, according to the characteristics of the shaft oscillation signal, the time-domain handcrafted features were selected as RMS and peak value. The frequency-domain handcrafted features were selected as the amplitude at the rotating frequency (1f), the amplitude at the double frequency (2f), the amplitude at the triple frequency (3f), the amplitude at the quadruple frequency (4f), the amplitude sum in the frequent band [0, 0.5f], the amplitude sum in the frequent band [0.5f, 1f], the amplitude sum in the frequent band [1f, 2f], and the amplitude sum in the frequent band [2f, 5f].

**Figure 6:**
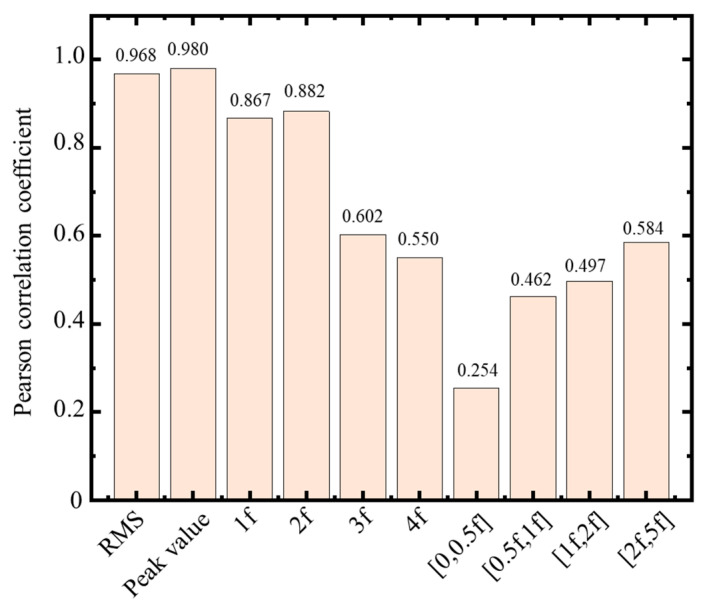
Pearson correlation coefficient of time- and frequency-domain handcrafted features for the unbalanced force.

Finally, we conducted a Pearson correlation analysis on the handcrafted features. Figure 6 shows the Pearson correlation coefficient for each feature with the unbalanced force. Since the RMS and peak value strongly correlated with the unbalanced force among the time- and frequency-domain handcrafted features, a feasible explanation is that the hypergravity centrifuge is an arm-type rotor, and the vibration response contains more harmonic components of rotating frequency.

**Figure 7:**
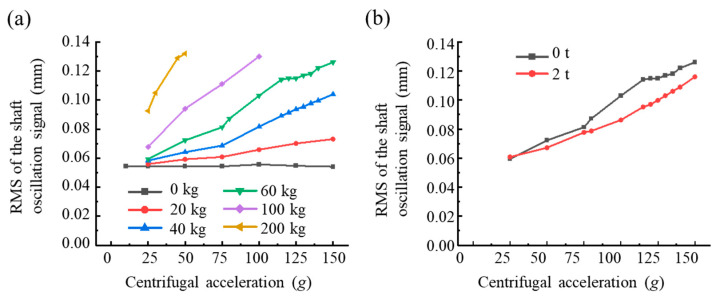
Effects of centrifugal acceleration on the RMS of shaft oscillation. (**a**) RMS of the shaft oscillation at different added unbalanced masses (0 t operating load). (**b**) RMS of the shaft oscillation at 0 t and 2 t operating loads (60 kg added unbalanced mass).

Besides the time- and frequency-domain vibration signal features, the operating state of the hypergravity centrifuge, i.e., the centrifugal acceleration and the operating load, affected the unbalanced force. Figure 7a illustrates the effects of centrifugal acceleration and added unbalanced mass on shaft oscillation in the unbalance response test. For the same unbalanced mass, the RMS of the shaft oscillation approximately increased linearly with increasing centrifugal acceleration. As shown in Figure 7b, we observed complexity in the operating load effect on shaft oscillation: the overall shaft oscillation trend decreased as the operating load increased but the decline varies at different centrifugal accelerations. Thus, we selected the centrifugal acceleration and operating load as the handcrafted features to improve the performance of unbalanced force identification.

### 3.3. Features Normalization Processing

Finally, the handcrafted features were normalized to eliminate the order of magnitude differences in the handcrafted features and reduce the computation time, as shown below:

(11) $$x_{s}=\frac{x-\mu_{x}}{\sigma_{x}},$$

where $\mu_{x}$ and $\sigma_{x}$ are the mean and STD of x, respectively.

## 4. Model Evaluation

### 4.1. Evaluation Criteria for Model Assessment

Performance evaluations are designed to reveal the accuracy of the developed identification model. Therefore, we selected the MAE, root mean square error (RMSE), median absolute error (MedAE), explained variance regression score (EVS), mean Tweedie deviance regression loss (MTD), and coefficient of determination (R^2^) as six performance indicators to prove the correspondence between the identified and actual unbalanced force. The MAE, RMSE, MedAE, EVS, MTD, and R^2^ are defined as follows:

(12) $$\mathrm{MAE}=\frac{1}{N}\sum_{i=1}^{N}\left|y_{iF}-{\hat{y}}_{iF}\right|,$$

(13) $$\mathrm{RMSE}=\frac{1}{N}\sqrt{\sum_{i=1}^{N}{\left(y_{iF}-{\hat{y}}_{iF}\right)}^{2}},$$

(14) $$\mathrm{MedAE}=\mathrm{Median}\left\{\left|y_{1F}-{\hat{y}}_{1F}\right|,\ldots ,\left|y_{iF}-{\hat{y}}_{iF}\right|\right\},$$

(15) $$\mathrm{EVS}=1-\frac{\mathrm{Var}\{y_{F}-{\hat{y}}_{F}\}}{\mathrm{Var}\{y_{F}\}},$$

(16) $$\mathrm{MTD}=\frac{1}{N}\sum_{i=1}^{N}\left\{\begin{array}{ll} {\left(y_{iF}-{\hat{y}}_{iF}\right)}^{2}, & \;\mathrm{for}\;\rho =0\;(\mathrm{Normal})\;\\ 2\left(y_{iF}\log\left(y_{iF}/{\hat{y}}_{iF}\right)+{\hat{y}}_{iF}-y_{iF}\right), & \;\mathrm{for}\;\rho =1\;(\mathrm{Poisson})\;\\ 2\left(\log\left({\hat{y}}_{iF}/y_{iF}\right)+y_{iF}/{\hat{y}}_{iF}-1\right), & \;\mathrm{for}\;\rho =2\;(\mathrm{Gamma})\;\\ 2\left(\frac{\max{\left(y_{iF},0\right)}^{2-p}}{\left(1-p)(2-p\right)}-\frac{y_{iF}{\hat{y}}_{iF}^{1-p}}{1-p}+\frac{{\hat{y}}_{iF}^{2-p}}{2-p}\right), & \;\mathrm{otherwise}\; \end{array}\right.,$$

(17) $$R^{2}=1-\frac{\sum_{i=1}^{N}{\left(y_{iF}-{\hat{y}}_{iF}\right)}^{2}}{\sum_{i=1}^{N}\left(y_{iF}-{\bar{y}}_{iF}\right)},$$

where $y_{iF}$ indicates the actual unbalanced force of the *i*th sample, ${\hat{y}}_{iF}$ indicates the identified unbalanced force of the *i*th sample, N indicates the total number of samples considered, ${\bar{y}}_{iF}$ indicates the mean value of the actual unbalanced force among the samples considered, Median{⋅} and Var{⋅} indicate the median and variance operator, respectively. MAE reflects the average margin of error between the identified and actual values, RMSE describes the STD of the difference, MedAE and EVS reflect the median and variance, respectively, MTD reflects the difference according to Tweedie distribution, and ρ=1.5. The parameter R^2^ measures how well a model can replicate the observations.

### 4.2. Experimental Setup

The value of the power parameter ρ in Tweedie loss and the validity of the feature fusion framework was first verified to build the deep learning model. Next, to compare the performance of ResNet using Tweedie loss in the unbalanced force identification problem, we implemented ResNet with different loss functions, such as MAE, MSE, and Huber. We also compared the proposed approach and several widely used benchmark approaches, such as machine learning methods SVM, ETR, XGBoost, and deep learning methods SENet, and MobileNet. Then, cross-validation was performed with the training dataset to determine the parameters of the above approaches. The initial learning rate was 0.001, the dropout rate was 0.4 for 200 iterations, the ResNet was ResNet-50, the SENet was SE-ResNet-18, and the MobileNet was MobileNetV1.

## 5. Results and Discussion

### 5.1. Model Development and Parameter Optimization

The ResNet-Tweedie algorithm model was developed using the approaches proposed in Figure 2. However, to improve reliability, the value of the power parameter ρ and the validity of the feature fusion framework had to be set: whether using the feature fusion framework (True or False) or the power parameter ρ (separately set to 1.1, 1.3, 1.5, 1.7, 1.9), with each algorithm running five times for the training dataset. Table 3 reports the average indices and relevant ranking results. Investigations revealed that although the feature fusion framework had no significant effect on the MAE index, and the RMSE index has significant advantages, the effect pattern of the power parameter on the MAE and RMSE indices was complicated. Hence, the best combination of parameters was determined by combined ranking (using the feature fusion framework and ρ=1.7).

### 5.2. Performance Analysis of the Unbalanced Force Identification Model

**Figure 8:**
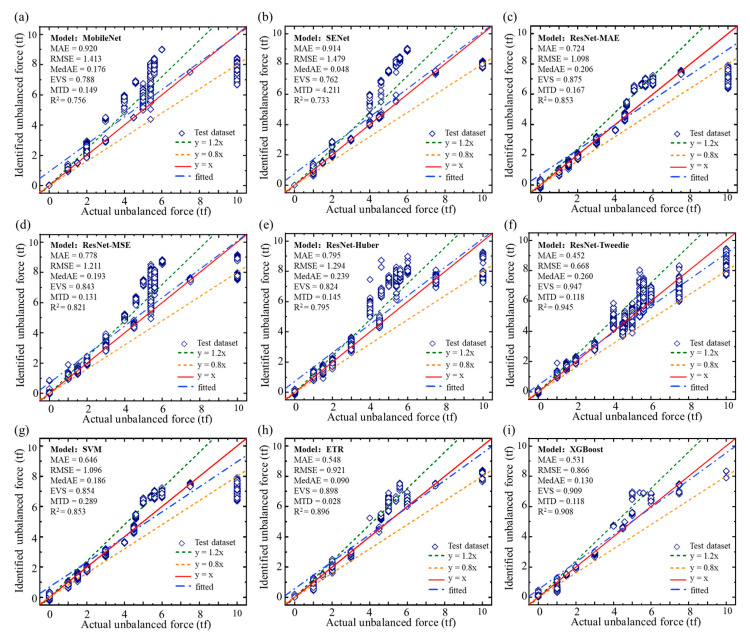
The unbalanced force identification results of each model. (**a**) MobileNet; (**b**) SENet; (**c**) ResNet-MAE; (**d**) ResNet-MSE; (**e**) ResNet-Huber; (**f**) ResNet-Tweedie; (**g**) SVM; (**h**) ETR; (**i**) XGBoost.

Figure 8 compares the unbalanced force identification results with some widely used benchmark approaches and the actual unbalanced force is plotted together as a criterion. The identification results are fitted by linear regression.

Table 4 shows the performance of each model analyzed by the six indicators (MAE, RMSE, MedAE, EVS, MTD, and R^2^). When machine learning methods, such as SVM, ETR, and XGBoost were used for unbalanced force identification, the MAE and RMSE indicators of the test dataset identification results showed advantages in comparing several deep learning methods without considering sample imbalance. We observed limited generalization ability in several machine learning approaches, i.e., SVM and ETR, all the machine learning models had significant identification errors for the large, unbalanced forces (greater than 9 tf). Furthermore, sample imbalance affected deep learning approaches more severely than machine learning approaches. Among them, ResNet-Tweedie had the best generalization capabilities, allowing capturing of complex nonlinear relationships between the input and output variables. Notably, using Tweedie loss as the loss function can optimize the sample imbalance problem and improve the identification capability of the model. Figure 8 and Table 4 show that the ResNet-Tweedie model performed better in critical indicators MAE, RMSE, EVS, and R^2^. Thus, the proposed approach outperformed all benchmark approaches.

### 5.3. Analysis of Identification Errors with Box-Plot

**Figure 9:**
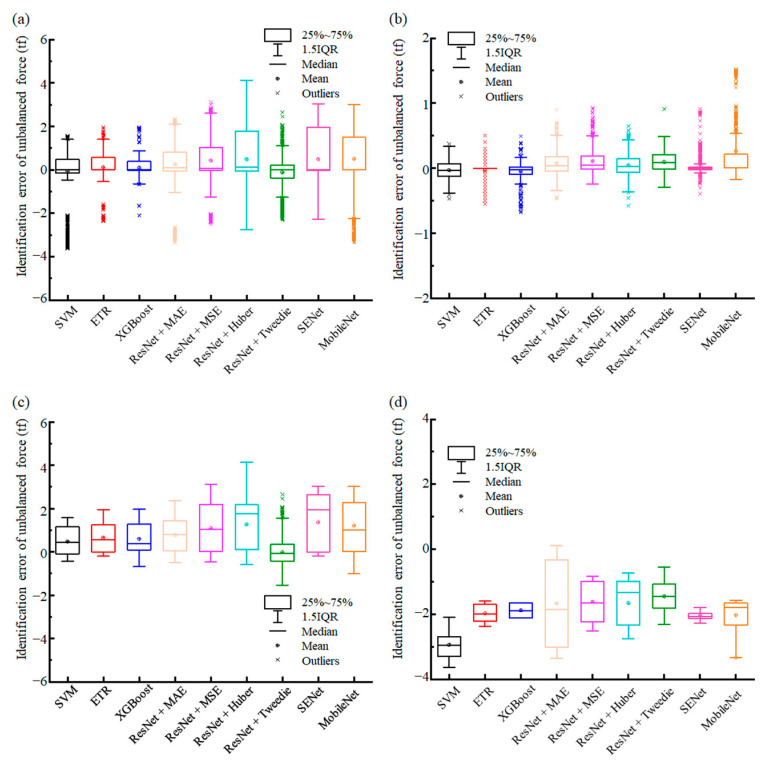
The identification error of each model’s test dataset. (**a**) Entire test dataset; (**b**) Many-shot region; (**c**) Few-shot region; (**d**) Missing-shot region.

Figure 9 shows the identification error in the test dataset of the unbalanced force identification model for the hypergravity centrifuge used in this paper. The identification error was defined as:

(18) $$\mathrm{error}={\hat{y}}_{F}-y_{F},$$

where ${\hat{y}}_{F}$ is the unbalanced force identification result, and $y_{F}$ is the actual unbalanced force.

Figure 9a illustrates the error for the entire test dataset and that the results identified by different models were all approximately equal to 0. The SVM, ETR, XGBoost, and ResNet-Tweedie models performed better in the error distribution range. Subsequently, Figure 9b–d show the errors of these three regions. The performances of each model were similar, with high identification accuracies in the Many-shot region. In addition, the identification performance of the ResNet-Tweedie model in the Few-shot region was significantly advantageous over the other comparative models. The performance of the ResNet-Tweedie model was effectively improved in the Few-shot region, owing to the proposed Tweedie loss. Deep learning models have greater potential than machine learning models for the identification of extremely large, unbalanced forces. It is also noteworthy that while the performance of each comparison model was weak in the Missing-shot region, the probability of such a massive, unbalanced force in the Missing-shot region was low in practice, probably occurring in a state of very high centrifugal acceleration or severe counterweight error. Thus, we propose to improve the identification performance in the Missing-shot region by expanding the sample.

### 5.4. Balance Status Identification and Counterweight Evaluation during the Speed-Up Process

Hypergravity centrifuges often set several transition centrifugal accelerations during the speed-up process, which slowly increases the acceleration stepwisely until the design acceleration of the hypergravity test is reached. Consequently, we can obtain sensor signals at multiple transition centrifugal accelerations and identify the corresponding unbalanced forces by the unbalanced force identification method, and calculate the unbalanced mass according to Equation (10).

**Figure 10:**
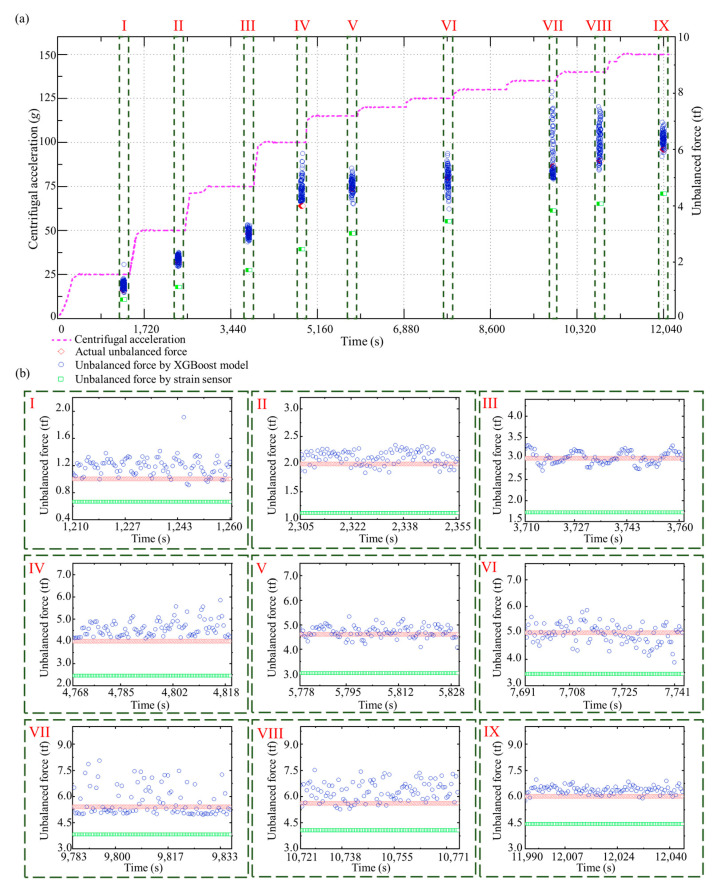
Unbalanced force identification results with the ResNet-Tweedie model. (**a**) The whole speed-up process. (**b**) Identification results during each centrifugal acceleration step.

**Figure 11:**
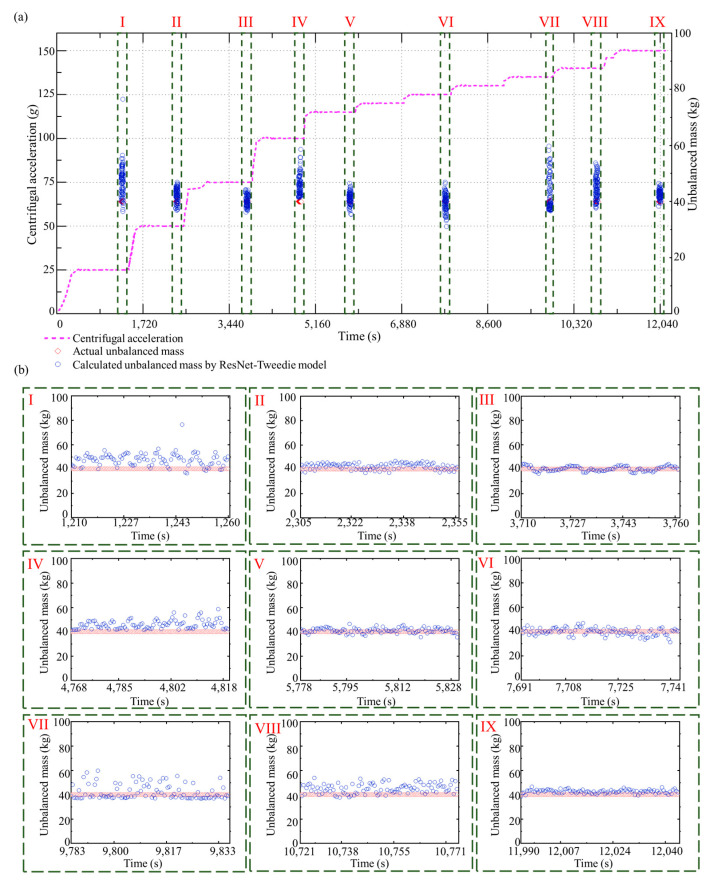
Unbalanced mass calculation results with the ResNet-Tweedie model. (**a**) The whole speed-up process. (**b**) Identification results during each centrifugal acceleration step.

**Figure 12:**
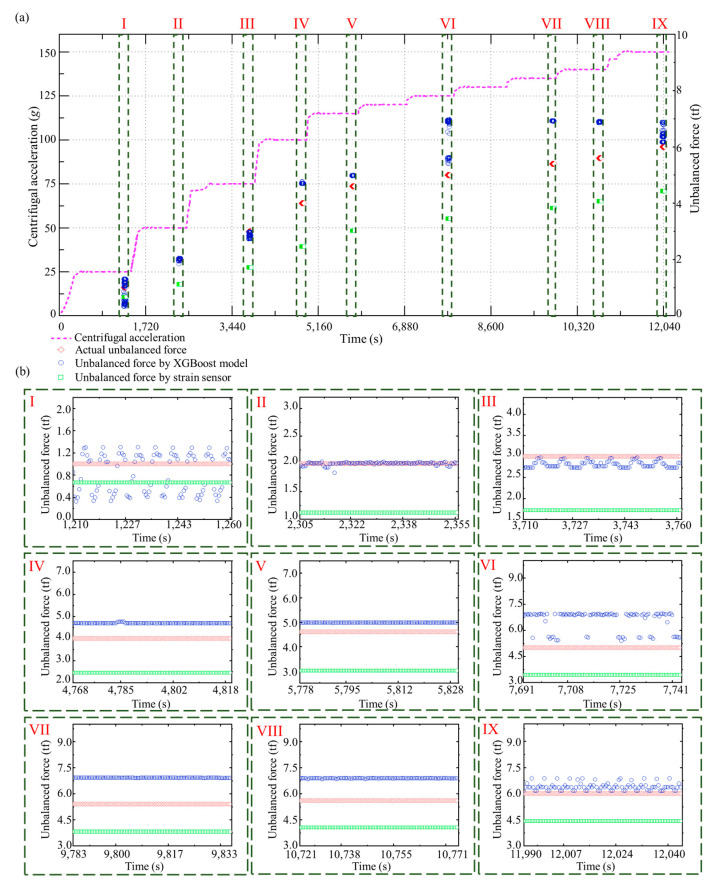
Unbalanced force identification results with the XGBoost model. (**a**) The whole speed-up process. (**b**) Identification results during each centrifugal acceleration step.

**Figure 13:**
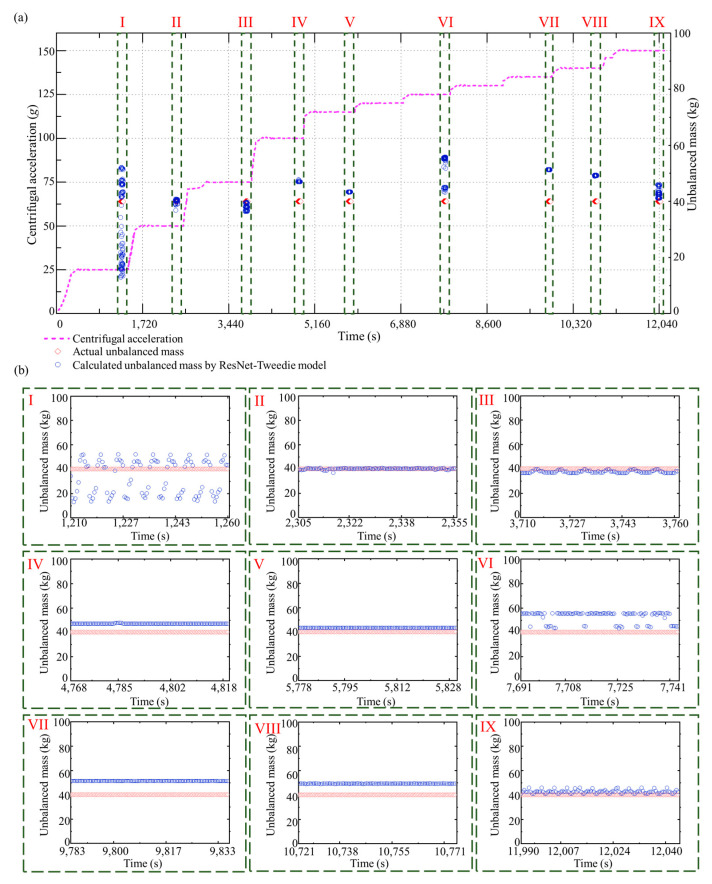
Unbalanced mass calculation results with the XGBoost model. (**a**) The whole speed-up process. (**b**) Identification results during each centrifugal acceleration step.

In the test dataset, an entire speed-up process was selected for analysis, in which the designed centrifugal acceleration was 150 *g*, and the added unbalanced mass was 40 kg. Furthermore, we installed strain sensors in the hypergravity centrifuge to indirectly measure the unbalanced force by evaluating the deformation of the rotational arm. The results of the unbalanced force identification using the ResNet-Tweedie model and strain sensors for this speed-up process are shown in Figure 10a. Multiple steps of centrifugal acceleration can be observed during the speed-up process. The unbalanced force identification results when the centrifugal acceleration reaches 25 *g*, 50 *g*, 75 *g*, 100 *g*, 115 *g*, 125 *g*, 135 *g*, 140 *g*, and 150 *g*, are shown in Figure 10b, respectively. The unbalanced force increases with the increasing centrifugal acceleration. The MAE, STD, and median of the absolute errors of the proposed method are 0.35 tf, 0.38 tf, and 0.24 tf. As a comparison, these three metrics of identification method with strain sensors are 1.41 tf, 0.38 tf, and 1.55 tf. Even though recognition performance varies at different centrifugal accelerations, the proposed method in this paper outperforms current methods by 75% in accuracy and by 85% in stability. The calculated unbalanced masses during the speed-up process and each centrifugal acceleration step are shown in Figure 11a,b, respectively. The calculated unbalanced mass result is close to the artificially added mass of 40 kg during the whole speed-up process (MAE is 3.80 kg). In order to highlight the advantages of proposed deep learning methods, the speed-up process is also investigated using XGBoost. Figure 12 and Figure 13 show the result of unbalanced force and unbalanced mass by XGBoost, respectively. It can be observed that at large centrifugal acceleration, the unbalanced force identification error using XGBoost is significant. The MAE, STD, and median of the absolute errors of XGBoost are 0.71 tf, 0.60 tf, and 0.47 tf. The proposed deep learning-based approach has advantages in accuracy and stability. As shown in Figure 13b, at partial centrifugal acceleration, the calculated unbalanced mass by XGBoost has severely deviated from 40 kg (MAE is 5.59 kg during the whole speed-up process), which may lead to incorrect guidance of the counterweight. Compared with the current method with strain sensors, the proposed method reduced the unbalanced force identification error by about 75% and the median by about 85%. Therefore, the ResNet-Tweedie model performed better and effectively evaluated the hypergravity centrifuge’s balance status, guiding the balancing counterweight.

## 6. Conclusions

This paper proposed a deep learning-based unbalanced force identification of hypergravity centrifuges via a procedure based on artificially added unbalanced masses to obtain the shaft oscillation signal of a hypergravity centrifuge under unbalanced forces. Then, a feature fusion framework was developed, which combined time-domain signal features extracted by ResNet and meaningful handcrafted features, followed by combining the feature fusion framework with Tweedie loss to investigate the identification effect of using this model, solving the issue of the imbalanced dataset. Compared with the traditional unbalanced force identification method in rigid rotor systems, the proposed approach bypasses trial weight and complex modeling, and can therefore be directly applied to hypergravity centrifuges. Finally, the developed model was compared with several widely used benchmark models in accuracy and generalization, then applied to the test data of the ZJU-400 hypergravity centrifuge. The following conclusions were drawn:

1. The identification results of the ZJU-400 hypergravity centrifuge after training showed that the unbalanced force identification model proposed in this paper could accurately extract features from the shaft oscillation signal and reveal the complex relationship between features and unbalanced force. Consequently, the MAE, RMSE, and R^2^ of the test dataset were 0.452 tf, 0.668 tf, and 0.945, respectively. The proposed method obtained the best performance among the benchmark deep learning and machine learning methods. Specifically, it reduced the MAE by 15%~51% and the RMSE by 22%~55% compared with other benchmark methods, illustrating its high identification accuracy and stability. The model proposed in this paper also met the demand for quantitatively identifying unbalanced forces in hypergravity centrifuges without trial weight.
2. According to the research results, Tweedie loss improved the identification performance in imbalanced datasets that exhibited long-tailed distributions, indicating a significant reduction in the identification error of large, unbalanced forces.
3. During centrifuge speed-up, identification accuracy and stability were judged by assessing the consistency of the calculated unbalanced mass, and the correlation between the identified unbalance force and centrifugal acceleration. Accordingly, the unbalanced force identification method proposed in this paper achieved accurate identification and provided an evaluation for balancing counterweight in the centrifuge speed-up process, surpassing the strain sensor-based method by 75% in the MAE (accuracy), and by 85% in the median (stability).

The deep learning-based unbalanced force identification approach proposed in this paper accurately and quantitatively identified the balance status of the hypergravity centrifuge, provided guidance for counterweight, and guaranteed the stability/safety of the unit. Hence, the methodology possesses the potential for the quantitative unbalance identification of general rotor systems without trial weight.

## Figures and Tables

**Table 1:** Experimental conditions and unbalanced force range for the training dataset.

	Centrifugal Acceleration (*g*)	Operating Load (t)	Unbalanced Mass (kg)	Unbalanced Force (tf)
Part 1	25, 50, 100, 125, 150	0, 2	0, 20	0–3
Part 2	25, 50, 100, 115, 125, 135, 140, 150	0	40	1–6
Part 3	25, 50, 80, 100, 125, 135, 140, 150	0, 2	60	1.2–9
Part 4	25, 50	0, 2	100	2.5–5
Part 5	25, 30, 45	0	200	5–9
Part 6	25, 30, 40	2	200	5–8

**Table 2:** Experimental conditions and unbalanced force range for the test dataset.

	Centrifugal Acceleration (*g*)	Operating Load (t)	Unbalanced Mass (kg)	Unbalanced Force (tf)
Part 1	25, 50, 100, 115, 125, 135, 140, 150	2	40	1–6
Part 2	75	0, 2	0, 20, 40, 60, 100	0–7.5
Part 3	50	0, 2	200	10
Part 4	100	0, 2	100	10

**Table 3:** The performance after optimizing ResNet-Tweedie.

ρ	Whether Using the Feature Fusion Framework	MAE	Rank	RMSE	Rank	Total
1.1	True	0.045	9	0.38	9	18
1.3	True	0.034	5	0.30	5	10
1.5	True	0.025	3	0.28	4	7
1.7	True	0.024	2	0.26	2	**4**
1.9	True	0.035	6	**0.25**	1	7
1.1	False	0.048	10	0.38	9	19
1.3	False	0.038	7	0.26	2	9
1.5	False	0.025	3	0.31	6	9
1.7	False	**0.022**	1	0.36	8	9
1.9	False	0.040	8	0.32	7	15

**Table 4:** The identification results of each model.

Model	MAE	RMSE	MeAE	EVS	MTD	R^2^
MobileNet	0.920	1.413	0.176	0.788	0.149	0.756
SENet	0.914	1.479	0.048	0.762	4.211	0.733
ResNet-MAE	0.724	1.098	0.206	0.875	0.167	0.853
ResNet-MSE	0.778	1.211	0.193	0.843	0.131	0.821
ResNet-Huber	0.795	1.294	0.239	0.824	0.145	0.795
ResNet-Tweedie	**0.452**	**0.668**	0.260	**0.947**	0.118	**0.945**
SVM	0.646	1.096	0.186	0.854	0.289	0.853
ETR	0.548	0.921	**0.090**	0.898	**0.028**	0.896
XGBoost	0.531	0.866	0.130	0.909	0.118	0.908

## Data Availability

The data presented in this study are available on request from the corresponding author. The data are not publicly available due to privacy.

## Nomenclature

**List of symbols**	
Y	A random variable which obeys the Tweedie composite Poisson distribution
P	A random variable conforming to the Poisson distribution
λ	Mean parameter of N
$X_{i}$	The variable Y’s value
α	Shape parameter of Gamma distribution
γ	Parameter of Gamma distribution
a(⋅)	Known function 1
κ(⋅)	Known function 2
θ	Parameter defined on real number field
ϕ	Dispersion parameter
$\kappa^{\prime }(\theta )$	The first derivative of θ
$\kappa^{\prime \prime }(\theta )$	The second derivative of θ
ρ	Power parameter
$y_{i}$	Actual value
$\mu_{i}$	Regression value
F	Unbalanced force
m	Artificially added mass (unbalanced mass)
ψ	Centrifugal acceleration at the bottom of the hanging basket
r	Nominal rotational radius of the hypergravity centrifuge
π	Ratio of circumference to diameter
f	Rotating frequency
x	Feature dataset
$x_{s}$	Normalized feature dataset
$\mu_{x}$	The mean of x
$\sigma_{x}$	The standard deviation of x
$y_{iF}$	Actual unbalanced force of the *i*th sample
${\hat{y}}_{iF}$	Identified unbalanced force of the *i*th sample
N	The total number of samples considered
${\bar{y}}_{iF}$	The mean of the actual unbalanced force among the samples considered
Median{⋅}	Median operator
Var{⋅}	Variance operator
p	Tweedie power parameter
${\hat{y}}_{F}$	The unbalanced force identification result
$y_{F}$	The actual unbalanced force
**List of acronyms**	
FEM	Finite element method
SVM	Support vector machine
MTF	Markov transition field
GAF	Gramian angular field
ETR	Extreme tree regression
XGBoost	Extreme gradient boosting algorithm
CNN	Convolution neural network
DBN	Deep belief networks
ResNet	Deep Residual Network
Senet	Squeeze-and-excitation networks
ZJU	Zhejiang University
GASF	Gram angular sum field
GADF	Gram angular difference field
RBB	Residual building blocks
MAE	Mean absolute error
MSE	Mean squared error
RMS	Root mean square value
Xf	x times the rotating frequency
RMSE	Root mean square error
MedAE	Median absolute error
MTD	Mean Tweedie deviance regression loss
R^2^	Coefficient of determination
STD	Standard deviation
